# Unusual Posterior Wall Uterine Rupture with the Use of Misoprostol for Second Trimester Pregnancy Termination

**DOI:** 10.4314/ejhs.v32i1.23

**Published:** 2022-01

**Authors:** Yitbarek Fantahun Mariye, Eskinder Kebede Weldetensay, Weyesa Dribisa

**Affiliations:** 1 Department of Obstetrics and Gynecology, School of Medicine, Addis Ababa University

**Keywords:** Abortion, Case report, Misoprostol, Second trimester, Uterine rupture

## Abstract

Abortion is defined as the termination of pregnancy before the fetus is viable. It is one of the most commonly performed procedures in gynecological departments worldwide. Termination of pregnancy in second trimester is one of the greatest challenges because of multiple modes of termination options with their risks of complication and making it riskier than the first trimester termination. We report this case because of a rare occurrence of posterior wall rupture which would have led to grave complication if not anticipated and detected early.

## Introduction

Abortion is defined as the termination of pregnancy before the fetus is viable and second trimester abortion is pregnancy termination between 12 and 28 weeks of GA. Although most abortions are performed in the first trimester, 10–15% of pregnancy termination occurs in second trimester worldwide. As compared to first trimester, second trimester abortions are disproportionately contributing for maternal morbidity and mortality especially in low-resource countries where there is limited access to safe abortion care (1).

Informed written consent was obtained from the patient for treatment reporting.

## Case Presentation

A 23 years old Gravida 2 para 1 mother who doesn't remember her LNMP but GA calculated from 16 weeks Ultrasound was 26 weeks pregnant. She came referred with diagnosis of missed abortion. She had decreased fetal movement for the past 1 week. She had one normal vaginal delivery 4 years back with baby weighing 3.2 kg with no maternal or neonatal complications. She had no history of uterine, cervical or vaginal procedures.

SFH was 24 weeks and ultrasound showed singleton intrauterine pregnancy with absent FHB with fundal placenta. She was admitted to the hospital and management options for missed abortion discussed and the decision was made for termination of pregnancy with Vaginal Misoprostol 200 every 4 hours for a total of 5 doses.

**Figure 1:**
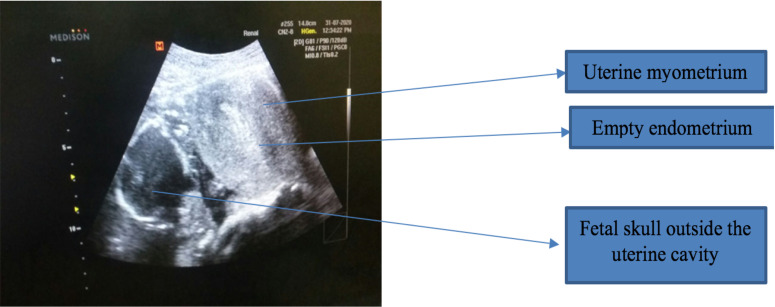
Ultrasound of the abdomen showing empty uterus with fetal parts in the abdominal cavity.

She was given a total of 5 doses and she started to have one uterine contraction every 10 min after the fifth dose of Misoprostol and 8 hours after the last dose she started to complain more persistent suprapubic abdominal pain otherwise no vaginal bleeding. Upon evaluation she had BP of 139/90, pulse rate of 104, respiratory rate of 14. She had suprapubic tenderness for deep palpation otherwise no signs of fluid collection. On pelvic examination, Cervix is 3cm dilated with no blood on examining finger with bulged posterior cul-desac. Obstetric Ultrasound done and it showed 16 x 10cm sized empty uterus with thick endometrial slit of 1.5cm with Intact gestational sac with conceptus tissue seen outside the uterus posteriorly. There was minimal fluid in the cul-de-sac (Figure 1).

**Figure 2:**
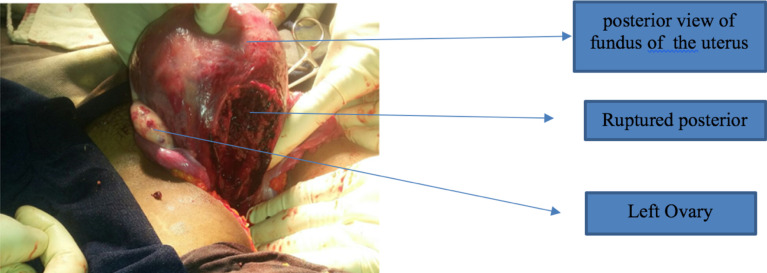
Intraoperative finding of posterior vertical uterine rupture.

After written informed consent taken patient was taken to the OR and abdominal cavity entered via midline infra-umbilical incision. Intraoperatively there was 8 cm posterior linear uterine rupture from lower segment extending to the fundus with intact gestational sac located posterior to the uterus. There was 400 ml of clot in the cul-de-sac with minimal bleeding from the rupture edges (Figure 2). Tubes and ovaries were healthy looking bilaterally. After the fetus and placenta removed and hemoperitoneum sucked out, the uterus repaired in three layers with Vicyl no. 1 and abdomen closed layer by layer.

## Discussion

Uterine rupture is an uncommon, but a life-threatening complication following second trimester medical termination of pregnancy in an unscarred uterus. The general occurrence is about 0.2% in the intact uterus and 3.8 to 4.3% in the scarred uterus (2).

The true incidence is not well known because of use of multiple modalities of termination and when complications happen, they are extremely rare but life-threatening and lethal if not well managed. Studies has showed an overall uterine rupture rate of 0.05% after misoprostol and 0.11% after surgical abortion among women with an unscarred uterus. Factors associated with rupture of an unscarred uterus are grandparity, placenta percreta, uterine anomalies, the dosing of misoprostol and concomitant use of oxytocine (3). There are multiple reports of Anterior and lateral report and posterior uterine rupture is not commonly reported (4,5). Yet, this case represents a gravida II woman with no prior surgical history who received a World Health Organization-recommended dose of misoprostol (6). One mechanism proposed for this type of rupture is the presence of both an uneffaced cervix and strong uterine contractions forcing the abortus through the lower posterior uterine wall instead of through cervix. Our patient did have an uneffaced cervix noted on examination upon evaluation.

Misoprostol is an acceptable choice for abortion induction in women in the second trimester; however, it is not risk-free. The complications though are uncommon but could be life threatening. There should always be a high index of suspicion and a close follow-up in women with second trimester abortion with misoprostol and the medical team should be well prepared to detect as well for managing life threatening complications to reduce the mortality in such case.
